# Ago2 protects *Drosophila* siRNAs and microRNAs from target-directed degradation, even in the absence of 2′-*O*-methylation

**DOI:** 10.1261/rna.078746.121

**Published:** 2021-06

**Authors:** Elena R. Kingston, David P. Bartel

**Affiliations:** Howard Hughes Medical Institute and Whitehead Institute for Biomedical Research, Cambridge, Massachusetts 02142, USA; Department of Biology, Massachusetts Institute of Technology, Cambridge, Massachusetts 02139, USA

**Keywords:** posttranscriptional regulation, endogenous siRNAs, siRNA dynamics, target-directed degradation, TDMD, RNA methylation

## Abstract

Target-directed microRNA (miRNA) degradation (TDMD), which is mediated by the protein ZSWIM8, plays a widespread role in shaping miRNA abundances across bilateria. Some endogenous small interfering RNAs (siRNAs) of *Drosophila* cells have target sites resembling those that trigger TDMD, raising the question as to whether they too might undergo such regulation by Dora, the *Drosophila* ZSWIM8 homolog. Here, we find that some of these siRNAs are indeed sensitive to Dora when loaded into Ago1, the Argonaute paralog that preferentially associates with miRNAs. Despite this sensitivity when loaded into Ago1, these siRNAs are not detectably regulated by target-directed degradation because most molecules are loaded into Ago2, the Argonaute paralog that preferentially associates with siRNAs, and we find that siRNAs and miRNAs loaded into Ago2 are insensitive to Dora. One explanation for the protection of these small RNAs loaded into Ago2 is that these small RNAs are 2′-*O*-methylated at their 3′ termini. However, 2′-*O*-methylation does not protect these RNAs from Dora-mediated target-directed degradation, which indicates that their protection is instead conferred by features of the Ago2 protein itself. Together, these observations clarify the requirements for regulation by target-directed degradation and expand our understanding of the role of 2′-*O*-methylation in small-RNA biology.

## INTRODUCTION

In metazoan systems, small RNAs direct processes that safeguard germline development, facilitate development of a variety of cell types and tissues, and maintain cellular homeostasis. These 20–30-nt RNAs are grouped into three major classes, distinguished by their biogenesis pathways: Piwi-interacting RNAs (piRNAs), small interfering RNAs (siRNAs), and microRNAs (miRNAs) (Malone and Hannon 2009; Bartel 2018; Czech et al. 2018). Small RNAs of all three classes associate with a member of the Argonaute (Ago) protein family. Acting as guide RNAs for their associated Ago proteins, the small RNAs regulate gene expression by pairing to sites within target transcripts to direct the repressive activities of the Ago proteins (or of their binding partners) to these complementary sequences, or in some cases, nearby chromatin (Höck and Meister 2008; Bartel 2009; Malone and Hannon 2009).

Expression levels and dynamics of a molecular species are specified by the interplay between the rates of production and degradation of that species. Although such rates have not been quantified for piRNAs and siRNAs, they have been elucidated for miRNAs in both mammalian and insect cells (Kingston and Bartel 2019; Reichholf et al. 2019). Most miRNAs are long-lived, presumably because Ago protects them from cytoplasmic nucleases, but some are turned over more rapidly. These rapidly turned-over miRNAs tend to be substrates of target-directed miRNA degradation (TDMD) (Shi et al. 2020), a phenomenon in which a target with unusually high complementarity to a miRNA drives turnover of that miRNA (Ameres et al. 2010; Cazalla et al. 2010). In the current model of TDMD, Ago–miRNA complexes associated with highly complementary targets are recognized by ZSWIM8, a substrate adaptor for a Cullin-RING E3 ubiquitin ligase (CRL), causing Ago to be degraded through the ubiquitin–proteasome system (Han et al. 2020; Shi et al. 2020). As Ago is degraded, the miRNA becomes exposed to cellular nucleases and is also degraded. In sum, more than 30 miRNAs have been implicated as TDMD substrates in mammalian cells, and analogous experiments have implied the presence of endogenous TDMD substrates in flies and worms, where ZSWIM8 is named Dora and Ebax-1, respectively (Shi et al. 2020). However, the degree to which other classes of small RNAs might too be regulated by this process is unknown.

*Drosophila* systems have long been used to understand the similarities and differences between siRNA and miRNA biogenesis and function, in part because siRNAs as well as miRNAs are highly expressed in the somatic cells of *Drosophila*, whereas siRNAs are not typically found in somatic cells of mammals. In *Drosophila*, these endogenous siRNAs are generated from Dicer-2 processing of long dsRNA substrates derived from either transposon RNAs, transcripts that fold back on themselves to form long hairpin structures, or paired regions of convergently transcribed transcripts (Chung et al. 2008; Czech et al. 2008; Ghildiyal et al. 2008; Kawamura et al. 2008; Okamura et al. 2008a,b). Dicer-1 processing generates miRNAs from shorter hairpin structures, and as during siRNA biogenesis, this processing produces ∼22-nt RNAs from both strands of the dsRNA substrate, which remain paired to each other with 2-nt 3′ overhangs (Zamore et al. 2000; Elbashir et al. 2001; Chung et al. 2008; Czech et al. 2008; Ghildiyal et al. 2008; Kawamura et al. 2008; Okamura et al. 2008b; Ha and Kim 2014). These short duplexes are then sorted into Ago1 or Ago2 on the basis of duplex complementarity and, to a lesser extent, 5′-nt identity (Okamura et al. 2004; Förstemann et al. 2007; Czech et al. 2009; Ghildiyal et al. 2010). Although these criteria cause most miRNAs to load into Ago1, some miRNAs are relatively enriched in Ago2, and conversely, although most siRNAs load into Ago2, some siRNAs are able to load into Ago1 (Okamura et al. 2004; Förstemann et al. 2007; Tomari et al. 2007; Czech et al. 2009; Ghildiyal et al. 2010; Ameres et al. 2011). Small RNAs loaded into Ago2 are subsequently methylated at the 2′-OH of their 3′-terminal nucleotide by the methyltransferase Hen1, which protects these small RNAs from tailing (the addition of nucleotides to the RNA 3′ end) and trimming (the removal of nucleotides from the RNA 3′ end) (Förstemann et al. 2007; Horwich et al. 2007; Czech et al. 2008; Kawamura et al. 2008; Ameres et al. 2010). Loss of Hen1 causes increased abundance of tailed and trimmed siRNAs as well as decreased abundance of select siRNAs (Okamura et al. 2008b; Ameres et al. 2010).

As is typical for metazoan systems, *Drosophila* miRNAs typically engage with targets through limited pairing near the 5′ end of the miRNA (known as the seed region), which directs degradation of mRNA targets with some translational repression also sometimes detected (Brennecke et al. 2005; Bartel 2009, 2018; Jonas and Izaurralde 2015; Agarwal et al. 2018). On the other hand, *Drosophila* siRNAs mediate repression by directing Ago2-catalyzed slicing of extensively complementary sites (Okamura et al. 2004; Förstemann et al. 2007). Whereas extensively complementary sites, similar to those that siRNAs engage with, can trigger TDMD of corresponding miRNAs, sites with limited complementarity to the 3′ region of the miRNA, such as those that miRNAs typically engage with, are incapable of triggering TDMD (Ameres et al. 2010; Baccarini et al. 2011; de la Mata et al. 2015; Sheu-Gruttadauria et al. 2019). Thus, the observation that 10 miRNAs are susceptible to TDMD in *Drosophila* S2 cells suggests that each of these miRNAs has at least one as-yet-unidentified highly complementary target site that triggers its degradation in these cells (Shi et al. 2020). In contrast to miRNAs, endogenous siRNAs are not thought to be susceptible to target-directed degradation (TDD), even though some pair to highly complementary target sites. Note that hereafter we use the TDD acronym to refer to target-directed degradation of either siRNAs or miRNAs. Note also that we restrict this term to conventional TDD, as genetically defined by its dependence on ZSWIM8/Dora, leaving open the formal possibility that other, yet-to-be-identified types of target complementarity might also destabilize siRNAs, miRNAs, or piRNAs.

siRNAs are proposed to be protected from TDD by the 2′-*O*-methyl modification that occurs at the 3′ terminus of each Ago2-associated small RNA (Förstemann et al. 2007; Horwich et al. 2007; Czech et al. 2008; Kawamura et al. 2008; Ameres et al. 2010). This proposal stems from an association between the process of TDD and increased tailing and/or trimming of the targeted small RNA (Ameres et al. 2010), which has since been observed for many additional TDD substrates (Baccarini et al. 2011; Marcinowski et al. 2012; Xie et al. 2012; de la Mata et al. 2015; Bitetti et al. 2018; Ghini et al. 2018; Kleaveland et al. 2018; Shi et al. 2020). Indeed, prior to the discovery of the role of the ZSWIM8 CRL in TDD, the working model of TDD has been that binding of the extensively paired target pulls the 3′ terminus of the small RNA from Ago, exposing it to tailing, which marks the RNA for trimming and ultimately degradation by cellular nucleases (Ameres et al. 2010; Sheu-Gruttadauria et al. 2019). Because the terminal 2′-*O*-methyl modification blocks tailing, under this model, siRNAs loaded into Ago2 and thus methylated at their 3′ ends would be recalcitrant to TDD, whereas any siRNAs loaded into Ago1 would be unprotected and subjected to removal from the cell via TDD (Ameres et al. 2010, 2011). The recent discovery of the role for the ZSWIM8 CRL in TDD decouples target-directed tailing and trimming (TDTT) from TDD (Han et al. 2020; Shi et al. 2020), raising new questions regarding the degree to which siRNAs might be regulated by ZSWIM8, and how methylation might influence with such regulation.

Here we use *Drosophila* S2 cells as a model system to investigate regulation of siRNAs by TDD. We observe TDD of both siRNAs and miRNAs loaded into Ago1 but not Ago2. Loss of methylation does not alter the susceptibility of Ago2-loaded small RNAs to TDD, suggesting that Ago2 itself offers protection from TDD. Supporting this model, TDD functions normally on methylated species in Ago1. Together, these findings elucidate what licenses a small RNA to be susceptible to TDD.

## RESULTS

### siRNAs and miRNAs in Ago2 are insensitive to TDD

Ten miRNAs are significantly up-regulated following CRISPR-mediated disruption of *dora* in S2 cells (Supplemental Fig. S1A; Supplemental Table S1; Shi et al. 2020), raising the question of whether this regulatory mechanism might extend to siRNAs also present in S2 cells. To focus on siRNAs, which are mainly loaded into Ago2 (Okamura et al. 2004; Förstemann et al. 2007; Tomari et al. 2007), we subjected small RNA samples to periodate oxidation and β-elimination prior to preparing libraries for small-RNA sequencing (sRNA-seq). This treatment takes advantage of the fact that most siRNAs are loaded within Ago2 and thus methylated at their 3′ termini (Horwich et al. 2007), which protects them from oxidation by periodate and subsequent β-elimination of the terminal nucleoside (Yang et al. 2007). In contrast, small RNAs loaded into Ago1, including most miRNA molecules, are unmethylated and thus vulnerable to periodate oxidation and β-elimination, which leaves a 3′ phosphate that blocks ligation of the adaptor used to prepare the sequencing libraries. Comparison of sRNA-seq libraries from treated and untreated samples confirmed enrichment of siRNAs in the treated sample (Supplemental Fig. S1B), and enrichment values for miRNAs that agreed well with previously published values (Supplemental Fig. S1C).

**FIGURE 1.**
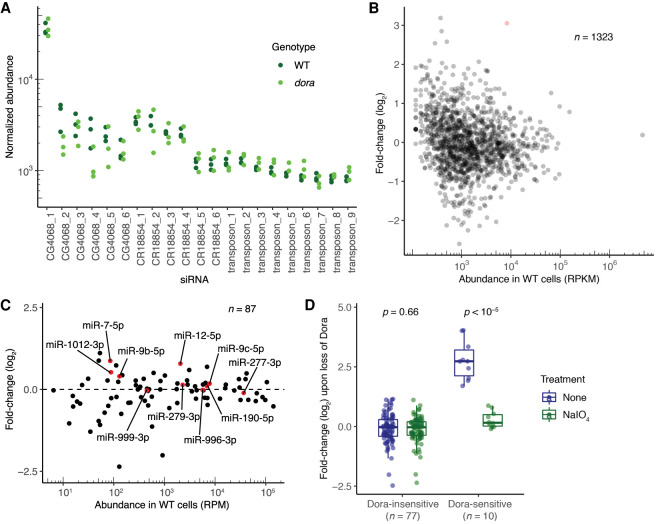
Methylated small RNAs are not susceptible to TDD. (*A*) Normalized abundance of the most highly expressed individual siRNAs in periodate-treated samples from wild-type (WT, dark green) and *dora* (light green) cells, as measured using sRNA-seq. Abundance is plotted as reads normalized to a cohort of abundant, Ago2-enriched, Dora-insensitive miRNAs (Supplemental Table S1). Shown are the results from three biological replicates for each genotype, each of which used a different clonal line. Significance was evaluated by a Welch two-sample *t*-test. (*B*) Changes in abundance of siRNAs mapping to annotated siRNA-generating loci observed upon loss of Dora in periodate-treated samples analyzed in *A*. Fold-changes in normalized abundance are plotted as a function of abundance observed in WT cells (RPKM, reads per kilobase per million mapped reads). Each point represents the mean from the three biological replicates, as determined by CuffDiff (Trapnell et al. 2012). The locus for which siRNAs significantly increased upon loss of Dora (CuffDiff adjusted *P* < 0.05) is indicated in red. (*C*) Changes in miRNA levels observed upon loss of Dora in periodate-treated samples analyzed *A* and *B*. Each point represents the mean from the three biological replicates, as determined by DESeq. Shown in red and labeled are results for Dora-sensitive miRNAs, identified as miRNAs that significantly increased (adjusted *P* < 2 × 10^–7^) upon loss of Dora in a different analysis that examined untreated, total-sRNA samples (Supplemental Fig. S1A; Shi et al. 2020). (*D*) Changes in miRNA levels observed upon loss of Dora, comparing results for total-sRNA (dark blue) and periodate-treated (dark green) samples for both Dora-sensitive and Dora-insensitive miRNAs. MicroRNAs were classified as Dora sensitive as in *C*. Significance was evaluated by a Welch two-sample *t*-test.

When comparing levels of the most highly expressed siRNA species in treated samples from wild-type S2 cells with those from *dora* clonal lines, no siRNA was significantly up-regulated upon loss of Dora (Fig. 1A; Supplemental Table S2). Although these 21 siRNA species are produced at a relatively high abundance, many siRNA species accumulate to much lower levels, as some siRNA-producing dsRNA regions are diced without a strongly stereotyped register (Chung et al. 2008; Czech et al. 2008; Ghildiyal et al. 2008; Kawamura et al. 2008; Okamura et al. 2008a,b). Thus, to extend our analyses to all siRNAs in S2 cells, we mapped small RNA reads from all treated samples to loci with siRNA-generating potential and examined siRNA levels from each of these loci, comparing levels in wild-type cells with those from *dora* clonal lines (Supplemental Table S3). siRNA reads mapping to all but one of these loci were unchanged upon loss of Dora (Fig. 1B). The one locus in which siRNA reads significantly increased upon loss of Dora (fold-change 7.5, *P* = 0.022), also had increased siRNA precursor upon loss of Dora as measured by RNA-seq, albeit with weak statistical significance (fold-change 9.2, unadjusted *P* = 0.17, Welch two-sample *t*-test), suggesting that the observed increase in siRNAs might be attributed to increased transcription rather than increased siRNA stability.

These results showing that siRNAs loaded into Ago2 do not detectably change upon loss of Dora indicated that either these siRNAs are not susceptible to TDD under native cellular conditions or these siRNAs each lack a target with complementarity suitable for triggering TDD. The latter seemed unlikely, as CG4068_1 (previously annotated esi-2.1), CG4068_5, and CG4068_6 exhibit high complementarity to a verified siRNA-sensitive, sliced site within the *mus308* mRNA (Czech et al. 2008; Okamura et al. 2008b). Moreover, many siRNAs from the CR18854 hairpin have highly complementary sites in the *CG8289* mRNA, although slicing at these sites has yet to be verified (Czech et al. 2008; Okamura et al. 2008b). To investigate whether small-RNA species loaded into Ago2 would be susceptible to TDD in the presence of sites that trigger TDD, we examined the fate of miRNAs in these periodate-treated samples. The 10 miRNAs that are significantly up-regulated upon loss of Dora in libraries generated from total-sRNA samples from S2 cells (henceforth referred to as the Dora-sensitive miRNAs) presumably have sites that trigger TDD (Supplemental Fig. S1A; Shi et al. 2020), yet none of these 10 miRNAs were significantly up-regulated upon loss of Dora in periodate-treated samples (Fig. 1C; Supplemental Table S1). When considered in the aggregate, the behavior of these Dora-sensitive miRNAs in the periodate-treated samples approached that of the group of Dora-insensitive miRNAs, with only a hint of a residual response to loss of Dora in the periodate-treated samples, which was attributable to slight contamination from Ago1-loaded species that were not fully removed by the periodate treatment (Fig. 1D). Thus, examination of miRNAs supported the conclusion that even in the presence of sites that trigger TDD, small RNAs within Ago2 are not susceptible to regulation by TDD.

### siRNAs in Ago1 are sensitive to TDD

The observation that miRNAs are sensitive to loss of Dora in total-RNA samples but not in Ago2-enriched samples suggested that Ago1-loaded species are privileged in their ability to be regulated by TDD. As some siRNAs can, albeit with low frequency, load into Ago1 (Czech et al. 2009; Ameres et al. 2011), we wondered whether looking at these Ago1-loaded species might reveal regulation of siRNAs by TDD that was undetectable in the Ago2-enriched samples.

**FIGURE 2.**
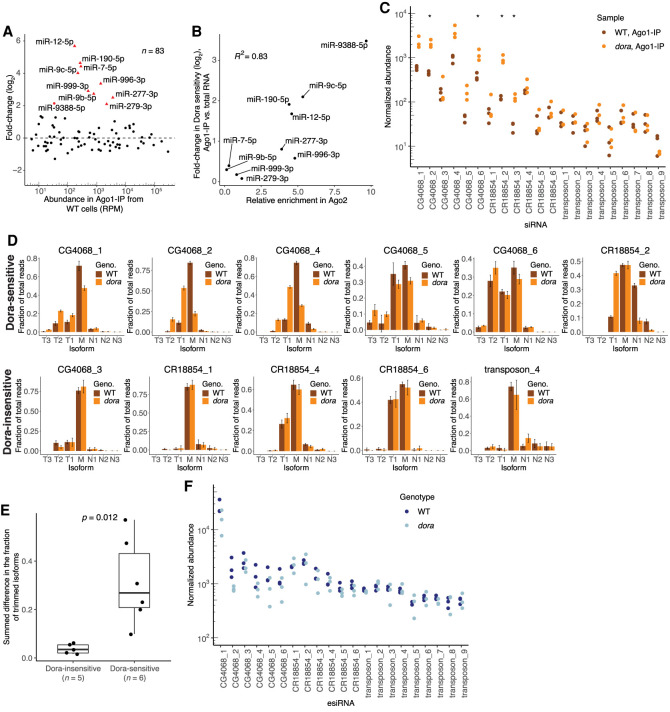
Small RNAs in Ago1 are susceptible to TDD. (*A*) Changes in levels of Ago1-associated miRNAs observed upon loss of Dora, as measured using sRNA-seq. Analysis was as in Figure 1C, but for miRNAs that copurified with Ago1. Results for miRNAs that significantly increased upon loss of Dora (adjusted *P* < 10^−20^) in the Ago1 samples are colored red and labeled, indicating with triangles those previously identified as Dora-sensitive in analyses of total-RNA samples (Supplemental Fig. S1A; Shi et al. 2020). (*B*) Relationship between the increased Dora sensitivity observed when examining Ago1-associated miRNAs and miRNA enrichment in Ago2. Results are shown for Ago1-associated miRNAs that most significantly increased upon loss of Dora (red points, *A*). Ago2 enrichment was inferred by dividing the average fraction of miRNA reads corresponding to that miRNA in the periodate-treated libraries by the average fraction of miRNA reads corresponding to that miRNA in the untreated libraries. The increased Dora sensitivity was computed by subtracting the log_2_-transformed fold-change observed upon loss of Dora in total-sRNA samples from the log_2_-transformed fold-change observed upon loss of Dora in the Ago1 samples. (*C*) Normalized abundance of the most highly expressed individual siRNAs in Ago1 samples from wild-type (WT, dark orange) and *dora* (orange) S2 cells, as measured using sRNA-seq. Significant differences are indicated (*) *P* > 0.05. Abundance is plotted as reads normalized to a cohort of abundant, Ago1-enriched, Dora-insensitive miRNAs (Supplemental Table S1); otherwise, this panel is as in Figure 1A. (*D*) Tailing and trimming of Dora-sensitive (*top* row) and Dora-insensitive (*bottom* row) siRNAs that passed the expression cutoff (an average of >40 reads across the wild-type samples) in Ago1 samples from wild-type (WT, dark orange) and *dora* (orange) S2 cells. Fractional abundance was quantified as the fraction of reads that the isoform contributed to the total reads for that siRNA and is shown for mature isoforms (defined as the most abundant isoform in wild-type cells), tailed isoforms with one to three additional nucleotides (N1, N2, N3), and trimmed isoforms with one to three fewer nucleotides (T1, T2, T3). Each of the two genotypes was represented by three clonal lines, and average fractional abundance and standard deviation (error bars) are shown for each isoform. (*E*) Changes in the fractions of trimmed isoforms observed upon loss of Dora for either the Dora-sensitive or Dora-insensitive siRNAs shown in *D*. Changes for each siRNA were quantified by summing the differences between the fractional abundances in *dora* and wild-type S2 cells for each trimmed isoform. Significance was evaluated by a Welch two-sample *t*-test. (*F*) Normalized abundance of the most highly expressed individual siRNAs in total-sRNA samples from wild-type (WT, dark blue) and *dora* (blue) S2 cells, as measured using sRNA-seq. Otherwise, this panel is as in *C*.

We utilized a FLAG-tagged fragment of human TNRC6B (the mammalian ortholog of GW182) to isolate Ago1-loaded small RNAs, which capitalized on the observation that GW182 strongly interacts with Ago1 but not Ago2 (Supplemental Fig. S2A,B; Behm-Ansmant et al. 2006; Hauptmann et al. 2015). We first verified Dora-mediated regulation of miRNAs in these samples and found that loss of Dora led to the significant increase of not only the 10 miRNAs that were previously found to be Dora-sensitive when examining small-RNA libraries made from total RNA but also miR-9388-5p (Fig. 2A), which had not been previously found to be Dora-sensitive (Supplemental Fig. S1A; Supplemental Table S1). Furthermore, the extent of Dora sensitivity was enhanced for most of the 10 previously annotated Dora-sensitive miRNAs (Fig. 2A,B), and this enhancement was highly correlated with enrichment in Ago2, with species that were more enriched in Ago2 exhibiting correspondingly greater Dora sensitivity in the Ago1-immunoprecipitate (Ago1-IP) samples (Fig. 2B). These results would be expected if miRNA molecules loaded into Ago1 were subjected to TDD, whereas those loaded into Ago2 were not, such that the full effects of TDD on Ago1-loaded miRNAs were only observed after removal of the TDD-non-responsive, Ago2-loaded population. Thus, our examination of miRNAs corroborated our previous assertions that small RNAs associated with Ago1 but not with Ago2 can be regulated by TDD.

The increased Dora sensitivity observed for Ago2-enriched miRNAs in the Ago1-IP samples encouraged us to look at siRNAs in these samples. Among the 21 most abundant siRNAs analyzed previously, four were significantly up-regulated upon loss of Dora (*P* < 0.05), and three others were up-regulated with *P*-values >0.05 but <0.075 (Fig. 2C; Supplemental Table S2). These seven Dora-sensitive siRNAs came from two hairpin loci (*CG4068* and *CR18854*, previously annotated as esi-2 and esi-1, respectively), but not all siRNAs produced from these two loci were sensitive to loss of Dora (Fig. 2C), presumably because some did not have target sites in the transcriptome with complementarity capable of triggering TDD. Three of the five *CG4068*-derived Dora-sensitive siRNAs have high complementarity to the validated targeted site in *mus308*, and one of the *CR18854*-derived Dora-sensitive siRNAs has high complementarity to a predicted targeted site in *CG8289* (Supplemental Fig. S2C; Czech et al. 2008; Okamura et al. 2008b). Interestingly, two other *CR18854*-derived siRNAs are also predicted to direct slicing of highly complementary sites in *CG8289* yet did not appear to be TDD substrates, perhaps because the siRNA seeds do not perfectly pair to the respective sites (Fig. 2C; Supplemental Fig. S2C). Highly complementary sites for the remaining Dora-sensitive siRNAs have yet to be identified.

Because complementary sites that direct miRNA degradation also promote tailing and trimming, the observation of increased tailing and trimming of Dora-sensitive miRNAs in the absence of Dora supports the proposal that these miRNAs each have complementary sites that trigger TDD in the presence of Dora (Shi et al. 2020). With respect to siRNAs, we found that compared to the Dora-insensitive siRNAs, Dora-sensitive siRNAs underwent increased trimming upon loss of Dora, thereby supporting the idea that these siRNAs also have complementary sites that direct degradation of Ago1-loaded molecules in the presence of Dora (Fig. 2D,E). Thus, taken together, our findings showed that siRNAs have the potential to be substrates of TDD but are protected from this degradation when associated with Ago2.

When Ago1-associated siRNAs were assessed more broadly by mapping reads to siRNA-generating loci, no widespread regulation by TDD was observed; siRNAs from only four loci significantly increased in abundance (including *CG4068* and *CR18854*) out of 697 examined (Supplemental Fig. S2D; Supplemental Table S3). Moreover, analysis of published data sets that sequenced total sRNAs (Shi et al. 2020) agreed with our analysis of small RNAs resistant to periodate oxidation (Fig. 1A), showing no detectable Dora sensitivity for any of the 21 highly expressed siRNAs (Fig. 2F). This inability to detect Dora sensitivity of siRNAs in total-sRNA samples was attributed to most of the siRNA signal for the total-sRNA sample coming from Ago2-loaded species, an assertion supported by the observation that the levels of most of the individual siRNAs were more than 10-fold lower in the Ago1-IP samples than in the total-RNA samples (Fig. 2C,F). Thus, although we observed robust TDD of select siRNA molecules that satisfied two criteria—association with Ago1 and complementarity to sites that seemed capable of triggering TDD—because relatively few siRNA molecules satisfied both of these criteria, the overall contribution of TDD to total siRNA levels was negligible.

### Methylation is not required to protect Ago2-loaded siRNAs from TDD

The observation that siRNAs are susceptible to TDD when loaded into Ago1 but not Ago2 indicated that some aspect of residing in Ago2 protects small RNAs from regulation by Dora. Ago2–siRNA complexes differ from Ago1–siRNA complexes with regard to both the protein and the siRNA. With respect to the protein, the *Drosophila* Ago1 and Ago2 proteins are quite divergent (26% sequence identity)—much more divergent than *Drosophila* Ago1 is with proteins that host small RNAs susceptible to TDD, such as human AGO2 (64% sequence identity). With respect to the siRNAs, the siRNAs within *Drosophila* Ago2 differ from those within Ago1 in that they are 2′-*O*-methylated at their 3′ termini (Förstemann et al. 2007; Horwich et al. 2007; Höck and Meister 2008; Kawamura et al. 2008; Okamura et al. 2008b). Although methylation was originally thought to protect small RNAs from TDD because it inhibits their tailing and trimming (Ameres et al. 2010), the recent discovery of ZSWIM8-mediated TDD, which operates independently from tailing and trimming, calls this proposal into question (Han et al. 2020; Shi et al. 2020).

**FIGURE 3.**
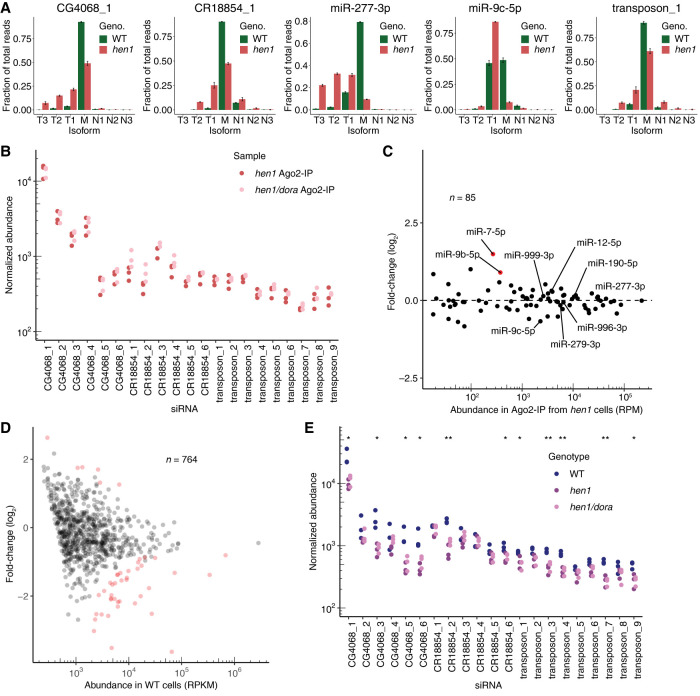
Loss of methylation does not alter sensitivity to TDD. (*A*) Tailing and trimming of Ago2-associated small RNAs from wild-type (WT, green) and *hen1* (red) S2 cells. Ago2-associated small RNAs were enriched by either periodate treatment (WT) or co-IP with FLAG-Ago2 (*hen1*) (Supplemental Fig. S3B). Otherwise, this panel is as in Figure 2D. (*B*) Normalized abundance of individual Ago2-associated siRNAs from *hen1* and *hen1/dora* cells, as measured using sRNA-seq. Ago2-associated siRNAs were isolated by IP of FLAG-Ago2. Otherwise, this panel is as in Figure 1A. (*C*) Changes in levels of Ago2-associated miRNAs observed upon loss of Dora in *hen1* cells. Ago2-associated miRNAs were isolated by IP of FLAG-Ago2. Each point represents the mean from three biological replicates, as determined by DESeq. Results for miRNAs sensitive to loss of Dora in wild-type cells are labeled (Supplemental Fig. S1A; Shi et al. 2020), and results for miRNAs significantly up-regulated (*P* < 10^−3^) upon Dora loss in these Ago2 samples are colored in red. (*D*) Changes in abundance of siRNAs mapping to annotated siRNA-generating loci observed upon loss of Hen1. Fold-changes in normalized abundance are plotted as a function of abundance observed in WT cells (RPKM, reads per kilobase per million mapped reads), as determined by CuffDiff. Differential expression was determined by DESeq, after normalizing to a cohort of abundant, Ago1-enriched, Dora-insensitive miRNAs (Supplemental Table S1). Each point represents the mean from the three biological replicates. The loci for which siRNAs significantly changed upon loss of Hen1 (DESeq adjusted *P* < 0.05) are indicated in red. (*E*) Normalized abundance of individual siRNAs in total-sRNA wild-type (WT), *hen1*, and *hen1/dora* samples. Note that the WT data are those of Figure 2D, replotted here for comparison, and that the normalization was performed as in Figure 2D. Significance was evaluated by ANOVA and the Tukey test.

To assess the relationship between methylation and the TDD pathway in a native context, we used Cas9 to generate clonal S2 cell lines that either lacked the methyltransferase Hen1 or lacked both Hen1 and Dora. Loss of Hen1 activity was confirmed by the increased sensitivity of both miR-277 and siRNA CG4068_1 to periodate oxidation and β elimination (Supplemental Fig. S3A). To evaluate the effect of Hen1 loss on Ago2-loaded species, we transfected a plasmid expressing FLAG-tagged Ago2 into the *hen1* and *hen1/dora* clonal lines and sequenced small RNAs that co-IPed with tagged Ago2. Enrichment for Ago2 was corroborated by western blotting (Supplemental Fig. S3B), and enrichment for Ago2-loaded miRNAs was corroborated by a high correlation of sRNA-seq results with results obtained from periodate-treated libraries, which substantially exceeded that observed when comparing to results obtained from total-RNA libraries (*R*^2^ = 0.91 and 0.69, respectively) (Supplemental Fig. S3C). In agreement with reports that Hen1 protects small RNAs from tailing and trimming (Li et al. 2005; Ameres et al. 2010; Kamminga et al. 2010, 2012; Montgomery et al. 2012), Hen1 loss caused increased tailing and trimming of Ago2-loaded small RNAs (Fig. 3A).

We next examined whether loss of Hen1 altered the susceptibility of Ago2-associated small RNAs to TDD. No significant up-regulation of any Ago2-loaded siRNA species was observed upon loss of Dora in the *hen1* background (Fig. 3B; Supplemental Fig. S3D; Supplemental Tables S2, S3). Furthermore, almost all Ago2-loaded miRNAs did not change in levels upon loss of Dora in the *hen1* background (Fig. 3C; Supplemental Table S1). The two exceptions were miR-7-5p and miR-9b-5p, which slightly increased upon Dora loss in the *hen1* background. These increases can be explained by the possibility of a small amount of contamination by Ago1-loaded species, which would most noticeably skew the results of these two Dora-sensitive miRNAs that were much more abundant in Ago1 than in Ago2. Thus, although select siRNAs and miRNAs were sensitive to loss of Dora when loaded into Ago1 (Fig. 2A,C), these same species escaped regulation by Dora when they were loaded into Ago2—even when lacking the terminal methyl modification (Fig. 3B,C). These results indicated that features of the Ago2 protein, not modifications to the RNA, protect Ago2-loaded small RNAs from TDD.

Previous studies of individual siRNAs report that loss of Hen1 can have a destabilizing effect (Okamura et al. 2008b; Ameres et al. 2011). To assess the consequence of Hen1 loss on siRNA levels globally, we compared total-sRNA samples from both wild-type and *hen1* cell lines. Reads mapping to 39 siRNA-producing loci significantly decreased, whereas reads mapping to six siRNA-producing loci significantly increased (Fig. 3D; Supplemental Table S3). Moreover, levels of 11 of the 21 most abundant individual siRNA species significantly decreased in the *hen1* cells, confirming observations of previous low-throughput studies (Fig. 3E; Supplemental Table S2). Importantly, however, none of these decreases were rescued by the additional loss of Dora (Fig. 3E).

When comparing the small-RNA reads observed with and without Hen1, an important consideration is that the methylation status of the small RNAs can bias adaptor ligation during preparation of sRNA-seq libraries (Munafó and Robb 2010), raising the possibility that the changes in sRNA-seq reads that we observed might not have fully reflected the changes in siRNA levels that occurred upon loss of Hen1. Arguing against this possibility, fold-changes determined by northern blotting agreed well with those determined by sequencing; by northern blotting, miR-277, CG4068_1, and CR18854_1 decreased by 43%, 53%, and 21%, respectively, whereas by sequencing, miR-277, CG4068_1, and CR18854_1 decreased by 38%, 64%, and 20%, respectively (Supplemental Fig. S3E). This agreement indicated that differential ligation efficiencies imparted minimal, if any, bias on our sRNA-seq results. Thus, taken together, our results show that loss of methylation by Hen1 alters the siRNA composition of S2 cells, destabilizing many siRNA species, but in a manner that occurs independently of the TDD pathway.

### Methylation does not protect an Ago1-loaded miRNA from TDD

**FIGURE 4.**
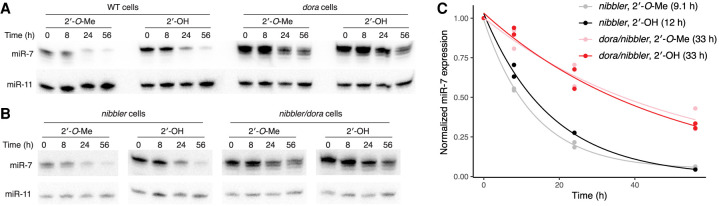
Methylation does not protect an Ago1-loaded miRNA from TDD. (*A*) Decay of methylated (2′-*O*-Me) or nonmethylated (2′-OH) miR-7 transfected into wild-type and *dora* cells. Decay was monitored on a northern blot probed both for miR-7 and endogenous miR-11, which served as a loading control. (*B*) As in *A*, but in the *nibbler* background. (*C*) Quantification of the results in *B* and those of one additional biological replicate, which used independent clonal cell lines (Supplemental Fig. S4E). The lines show the best least-squares fits of an exponential-decay model. Half-lives, indicated in parentheses, encapsulated the rates of both dilution and decay.

In human cells, methylation does not protect miRNAs from ZSWIM8-mediated decay (Han et al. 2020; Shi et al. 2020), which agrees with our finding that a terminal methyl modification is not required to protect Ago2-associated RNAs from TDD in S2 cells and raises the question as to whether methylation impacts TDD susceptibility of an Ago1-associated miRNA in *Drosophila*. To answer this question, we transfected either methylated or unmethylated miR-7 into wild-type and *dora* S2 cells, collected lysates of these transfected cells at a series of timepoints, isolated Ago1-associated species at each timepoint, and assessed miR-7 decay by northern blotting (Fig. 4A; Supplemental Fig. S4A,B). Methylation did not alter the susceptibility of miR-7 to TDD, in that increases in stability observed upon loss of Dora were similar for methylated and nonmethylated miR-7 (Supplemental Fig. S4C).

A caveat of these results, however, was that abundant trimmed isoforms of methylated miR-7 were observed, raising the possibility that this trimming, which removed the methylated nucleotide, might have influenced conclusions from this experiment. The exonuclease Nibbler is responsible for trimming the 3′ end of *Drosophila* miRNAs loaded into Ago1 (Han et al. 2011), which provided the means to address this concern by repeating the experiment in the *nibbler* background. Loss of Nibbler greatly reduced trimming of endogenous miR-7 and miR-34, yet did not impede TDD (Supplemental Fig. S4D), illustrating independence of the TDD and TDTT pathways. Furthermore, even in this *nibbler* background, methylated and unmethylated miR-7 had similar susceptibilities to TDD (Fig. 4B,C; Supplemental Fig. S4E). Thus, in *Drosophila* cells as in mammalian cells, methylation does not protect miRNAs from decay through the TDD pathway.

## DISCUSSION

Our analyses of siRNAs in both wild-type and *dora Drosophila* S2 cells demonstrated that this class of small RNAs undergoes little regulation by conventional TDD. This finding does not support the suggestion that siRNAs loaded into Ago1 instead of Ago2 might be “purified,” or removed from the cell, by TDD—a model put forth to help explain the high steady-state enrichment of siRNAs within Ago2 (Ameres et al. 2011). Although we do see TDD of some Ago1-loaded siRNAs, most siRNAs loaded in Ago1 escape such regulation (Fig. 2C; Supplemental Fig. S2D). Furthermore, even for the TDD-sensitive siRNAs, up-regulation upon loss of Dora was undetectable when examining total-sRNA samples (Fig. 2D), because, for each siRNA, the Ago1-loaded fraction was minimal when compared to the Ago2-loaded fraction, and thus any increase in the Ago1-loaded fraction (Fig. 2C) negligibly affected total siRNA levels. Thus, the known TDD pathway, which requires Dora, does not appear to be a major driving force in shaping the siRNA content of *Drosophila* S2 cells.

Although methylation of small RNAs is proposed to protect these RNAs from TDD (Ameres et al. 2010), we observed both that loss of methylation does not make Ago2-loaded species susceptible to the known TDD pathway and that gained methylation does not protect Ago1-loaded species from this pathway. These observations indicate that features of Ago proteins, rather than modifications of small RNAs, dictate the ability of Ago–RNA complexes to be regulated by TDD. This importance of the Ago protein concurs with the new model for TDD, in which Ago proteins must interact with and be ubiquitinated by the ZSWIM8/Dora CRL for TDD to occur (Han et al. 2020; Shi et al. 2020). Whereas Ago1 can engage with Dora in a TDD-competent manner, low sequence similarity between Ago2 and Ago1 supports the idea that Ago2 might lack the features necessary for Dora recognition and polyubiquitination. With respect to the sites of polyubiquitination, studies of human AGO2 implicate K493 and at least one other lysine within a cluster of 17 surface lysines as required for maximal ZSWIM8-mediated regulation (Han et al. 2020; Shi et al. 2020), of which K493 and 12 of the other candidates sites are conserved in *Drosophila* Ago1, whereas K493 and all but two of the other candidates sites are not conserved in *Drosophila* Ago2. By analogy, we speculate that if piRNAs are also protected from TDD, then this protection would also be conferred by the inability of PIWI proteins to interact with and be ubiquitinated by the ZSWIM8/Dora CRL.

Across many species, 2′-*O*-methylation occurs on guide RNAs that have extensive pairing to their targets, such as plant miRNAs (Yu et al. 2005), piRNAs (Ruby et al. 2006; Horwich et al. 2007; Kirino and Mourelatos 2007; Saito et al. 2007; Kurth and Mochizuki 2009; Kamminga et al. 2010, 2012; Billi et al. 2012; Montgomery et al. 2012), and some classes of endogenous siRNAs (Ruby et al. 2006; Horwich et al. 2007; Kawamura et al. 2008; Okamura et al. 2008b; Billi et al. 2012; Kamminga et al. 2012; Montgomery et al. 2012), but not on guide RNAs that lack extensive pairing to most of their targets, such as metazoan miRNAs (Bartel 2009). Loss of this methylation leads to increased tailing and trimming that, at least for plant miRNAs, Tetrahymena piRNAs, nematode 26G siRNAs, and some *Drosophila* siRNAs, is associated with small-RNA destabilization (Fig. 3A,D,E; Park et al. 2002; Li et al. 2005; Yu et al. 2005; Okamura et al. 2008b; Kurth and Mochizuki 2009; Ameres et al. 2010; Kamminga et al. 2010, 2012; Billi et al. 2012; Montgomery et al. 2012). The realization that the identity of the Ago protein rather than the methylation status of the small RNA dictates susceptibility to TDD reopens the mystery as to why the tendency to be methylated correlates with the degree of complementarity of typical sites for a given class of small RNA.

As a new solution to this mystery, we suggest that these classes of small regulatory RNAs with highly complementary sites reside in Ago/PIWI proteins that have intrinsically weaker interactions with the 3′ termini of their guide RNAs. This weaker intrinsic binding to guide-RNA 3′ termini is expected for these proteins because it would favor formation of extensive target pairing, as release of the 3′ terminus appears to be required to accommodate pairing to the central region of the guide RNA (Bartel 2018). In contrast, stronger intrinsic binding to guide-RNA 3′ termini is expected for proteins that associate with metazoan miRNAs, as release of the 3′ terminus is not required to accommodate target recognition typical of these small RNAs, i.e., seed pairing or seed pairing plus conventional 3′-supplementary pairing (Bartel 2018; Sheu-Gruttadauria et al. 2019). The weaker intrinsic binding proposed for Ago/PIWI proteins with guide RNAs that recognize highly complementarity sites would presumably leave the 3′ termini of their guide RNAs constitutively vulnerable to tailing and trimming even when they are not paired to a target, thereby explaining the benefit of terminal 2′-*O*-methylation. This Hen1-mediated methylation, found in plants and animals, presumably emerged early in eukaryotic evolution and thus would have been available for incorporation into the nascent metazoan miRNA pathway. Indeed, some methylation has been reported on most Nematostella miRNAs (Moran et al. 2014; Modepalli et al. 2018). Perhaps, however, as exemplified by the bilaterian lineage, as the miRNA-associated Ago proteins adapted to recognize less extensively paired sites, they acquired greater affinity to their guide-RNA 3′ termini, which reduced vulnerability to trimming and tailing thereby obviating a benefit for their methylation.

Several observations support aspects of our model. First, mutations within human Ago2 that reduce binding to miRNA 3′ termini promote tailing and trimming even in the absence of an extensively paired target (Sheu-Gruttadauria et al. 2019), which confirms the assumption that weaker binding to small-RNA 3′ termini imparts constitutive vulnerability to tailing and trimming. Second, the 3′ termini of piRNAs and metazoan siRNAs are 2′-*O*-methylated after these guide RNAs are loaded into Ago/PIWI (Horwich et al. 2007; Saito et al. 2007), implying that the methylation machinery has at least intermittent access to the guide-RNA 3′ termini, as would be expected if these proteins have relatively weak binding to the 3′ termini of their guide RNAs. Third, loss of Hen1 led to increased tailing and trimming of Ago2-associated siRNAs that were Dora-insensitive when associated with Ago1 (Fig. 3A, transposon_1), supporting the conjecture that Ago2-associated RNAs are vulnerable to tailing and trimming even in the absence of highly complementary sites able to trigger TDD.

The notion that small RNAs with 3′ termini not stably protected within Ago are susceptible to increased tailing and trimming might also help explain our observation that methylated miR-7, when loaded in Ago1, undergoes increased trimming relative to unmethylated miR-7 (Fig. 4A; Supplemental Fig. S4A,B). Perhaps the terminal methyl group is not well-accommodated by Ago1, which is typically loaded with unmethylated miRNAs, leading to increased exposure of the 3′ terminus of the methylated miR-7. Indeed, conformations of human AGO2 represented by the crystal structures would not accommodate a terminal methyl group, implying that terminal methyl modifications might similarly clash with the ground-state structure of *Drosophila* Ago1. Although methylation is thought to protect small RNAs from trimming in addition to tailing, the observation that trimming of methylated miR-7 is Nibbler sensitive suggests that, at least in the context of *Drosophila* Ago1, methylated species can still be trimmed.

In summary, the observation that some classes of small regulatory RNAs are methylated and some are not can be at least partly explained without invoking a TDD phenomenon: Because piRNAs, siRNAs, and plant miRNAs must efficiently pair to targets throughout their length, their corresponding Ago/PIWI proteins might have reduced affinity to guide-RNA 3′ termini, and this reduced affinity would render these guide RNAs more susceptible to tailing and trimming even when they are not paired to target—unless they are 2′-*O*-methylated.

## MATERIALS AND METHODS

### Cell culture, transfection, and gene editing

*Drosophila* S2 cells were grown at 26°C in Schneider's Drosophila Medium (Life Technologies) supplemented with 10% heat-inactivated FBS (Life Technologies). Cells were passaged 1:5 every 3–5 d. For transfections, cells were plated at a density of 2 million cells/mL, and then transfected with Effectene (Qiagen) according to the manufacturer's protocol. All cell counting was done using a Countess automated cell counter (Invitrogen). To generate knockout lines, cells were transfected with either one (for single-gene knockout) or two (for double knockout) cloned versions of pAc-sgRNA-Cas9 (Addgene #49330), constructed as per previously published protocols (Bassett et al. 2014) using oligonucleotides listed in Supplemental Table S5. Starting 3 d after transfection, cells were selected with 5 µg/mL puromycin (Life Technologies). After 1 wk of selection, single cells were sorted into wells to generate clonal lines. After culture for 2–3 wk, clonal lines were screened for lesions by PCR amplification of the targeted locus followed by TIDE analysis of the amplicon (Brinkman et al. 2014).

### RNA extraction

RNA was extracted with TRI Reagent (Life Technologies), according to the manufacturer's protocol. Briefly, samples were homogenized in TRI reagent, and RNA was phase-separated by the addition of chloroform (J.T. Baker Analytical) at a ratio of 250 µL per 1 mL TRI Reagent. RNA was then precipitated with isopropanol, with GlycoBlue (Life Technologies) added as a coprecipitant when necessary, and pellets were washed twice with 70% ethanol prior to resuspension in water.

### Transfection and analysis of synthetic miR-7

Synthetic miR-7 guide (both methylated and nonmethylated) and passenger strands (IDT, Supplemental Table S5) were purified on a 15% polyacrylamide urea gel, and resuspended in water at a final concentration of 10 µM. Equal volumes of guide and passenger strand were mixed with 2× Annealing Buffer (final concentration, 30 mM Tris, pH 7.5, 100 mM NaCl, 1 mM EDTA), and the mixture was heated to 90°C for 5 min before slow-cooling back to room temperature. Ten pmol of annealed duplex was transfected per well of a six-well plate, together with 1 µg of a GFP-expressing plasmid (a modified version of pAc-sgRNA-Cas9 designed not to express the sgRNA and to express HA-tagged GFP in place of Cas9) used as a carrier for the transfection. Cells were collected 24, 32, 48, and 80 h after transfection by resuspending the cells in their media, removing 1/4th of the resuspended cell mixture, and replacing the lost volume with fresh media. Collected cells were pelleted, and the cell pellets were snap frozen and stored at −80°C.

Ago1-associated small RNAs were isolated and analyzed by northern blotting, as described below. To calculate half-lives for the methylated and nonmethylated miRNAs, miR-7 levels were first normalized to those of miR-11. These values were then log-transformed and fit with a log-transformed single-exponential model of the form $y=\log(c_{2}e^{-c_{1}t}+c_{3})$, where *y* describes the miR-7:miR-11 ratio, *c*_*1*_ describes the half-life, *c*_*2*_ describes the initial miR-7:miR-11 ratio, and *c*_*3*_ describes the steady-state miR-7:miR-11 ratio (attributed to endogenous miR-7). Fitting was done in R, applying the optim function (method = “L-BFGS-B,” R v3.5.1) to minimize the sum of the squared residuals of the model to the data. The fit value for *c*_*2*_ was bounded to be between 0.001 and 10, and the fit values for *c_2_, and c*_3_ were bounded to be >10^−8^. To increase the chances that the true minimum was reached by the optim function, optimization was iterated 100 times for each data set using the parameter values reached by the previous round of optimization as the starting values for the next round. Half-lives were calculated as ln(2) divided by *c*_*2*_.

### sRNA-seq

Small-RNA libraries were generated as described in the step-by-step protocol available at http://bartellab.wi.mit.edu/protocols.html, with slight modifications. Briefly, between 5–10 µg of total RNA was mixed with size-selection markers (18 and 32 nt 5′-radiolabeled RNAs), and for the total-sRNA libraries quantitative sequencing standards were added (0.05 fmol per 1 µg total RNA). The samples were first size-selected, before undergoing subtractive hybridization to remove the 2S rRNA (Seitz et al. 2008). Degenerate adaptors (each containing four random-sequence nucleotides abutting the ligation junction) were then ligated to the 3′ and 5′ ends of the RNA in the presence of 10% Polyethylene Glycol (PEG 8000, NEB) and 0.5 µL of Superasin (Thermo Fisher Scientific) with T4 RNA Ligase 2, truncated K227Q (NEB) and T4 RNA Ligase 1 (NEB), respectively. Reverse transcription was carried out with SuperScript III (NEB), and the cDNA was PCR-amplified using Kapa HiFi HotStart Ready Mix (VWR). Libraries were sequenced on an Illumina HiSeq platform with 50-nt single-end reads. Oligonucleotides used for markers, subtractive hybridization, adaptors, and primers are listed (Supplemental Table S5).

### sRNA-seq analyses

Sequencing reads were processed by trimming the 5′ and 3′ ends using fastx_trimmer (FastX Toolkit; http://hannonlab.cshl.edu/fastx_toolkit/) and cutadapt (Martin 2011), respectively, and then filtering for greater than 99.9% accuracy for all bases using fastq_quality_filter (FastX Toolkit; http://hannonlab.cshl.edu/fastx_toolkit/) with the parameters “-q 30 –p 100”.

To call miRNA reads, the first 19 nt of filtered and trimmed reads were string-matched to a dictionary of miRNA sequences. The miRNA dictionary was curated by filtering miRbase_v21 miRNA annotations for all conserved or confidently annotated small RNA species (as annotated by TargetScanFly, release 7.2) as well as their passenger-strand partners. The few species that could not be called unambiguously using only the first 19 nt were collapsed into a single dictionary entry (the sequence of which was chosen randomly to be that of one of the collapsed species), which was listed under a merged name (for example, dme-miR-276a-5p and dme-miR-276b-5p became dme-miR-276ab-5p).

Differential expression of miRNAs was called using DESeq2 (R v3.5.1) (Love et al. 2014). For each plot, miRNAs were filtered to retain those with >5 RPM in Dora wild-type samples. MicroRNAs that varied dramatically in a single clone for a given genotype were removed. These outliers were identified as those for which the coefficient of variation of the standard-normalized (total-sRNA samples) or RPM-normalized (all other samples) for either genotype was more than 1.5 times the interquartile range below or above, respectively, the first or third quartile of the distribution of all of the coefficients of variation for all miRNAs in both genotypes. When examining Dora sensitivity, the significance threshold was the *P*-value below which there were only increases in abundance upon loss of Dora.

A dictionary of highly abundant individual siRNAs was curated by enumerating from the periodate-treated wild-type libraries the top 21 sequences that mapped to siRNA-generating loci. As with the miRNA analyses, reads corresponding to these individual siRNAs were identified by string-matching the first 19 nt of the filtered and trimmed reads to this dictionary. For cross-genotype comparisons of individual siRNAs from total-sRNA, Ago1-IP, and *hen1* samples, counts were normalized by the total number of reads assigned to a cohort of miRNAs that were Ago1-enriched (relative abundance in total-sRNA libraries versus sodium-periodate treated libraries <1; see below), abundant (average RPM > 1000 in total-sRNA libraries), and in aggregate underwent no change upon loss of Dora (Supplemental Table S1, norm1 column). For cross-genotype comparisons of individual siRNAs from periodate-treated and Ago2-IP samples, counts were normalized by the total number of reads assigned to a cohort of miRNAs that were similarly abundant and dora-insensitive but were instead Ago2-enriched (relative abundance in total-sRNA libraries versus sodium-periodate treated libraries >3; see below) (Supplemental Table S1, norm2 column).

For tailing and trimming analyses, suffixes to the 19 nt prefixes were enumerated for each small RNA. For each miRNA, the mature isoform was annotated as the species with the greatest number of reads in the wild-type, total-sRNA samples. For each siRNA, the mature isoform was annotated as the species with the greatest number of reads in the wild-type, periodate-treated samples. The tailed and trimmed species were then annotated with reference to this mature isoform; for these analyses, trimmed reads <19 nt were not considered, as counting required a string-match of the first 19 nt. For analyses comparing trimmed siRNA isoforms in Ago1-IP samples, only those siRNAs with an average of >40 reads in wild-type samples were used.

To count reads mapping to siRNA-producing loci, a list of unique, nonoverlapping regions in the genome that encompassed all previously published long hairpin loci, all transposons, and all regions of overlapping antisense-transcribed mRNAs (Chung et al. 2008; Czech et al. 2008; Ghildiyal et al. 2008; Kawamura et al. 2008; Okamura et al. 2008a,b; Watanabe et al. 2008) was manually curated (Supplemental Table S4). Any regions overlapping with an annotated miRNA transcript were removed from this list. Trimmed and filtered reads from small RNA libraries were then aligned to the genome (UCSC dm6.08 reference assembly) with STAR (V2.4, with the parameters “‐‐alignIntronMax 1 ‐‐outFilterMultimapNmax 50 ‐‐outFilterMismatchNoverLmax 0.04 ‐‐outFilterIntronMotifs RemoveNoncanonicalUnannotated ‐‐outSJfilterReads All”). Aligned reads mapping to siRNA-generating loci were then counted using cufflinks (v2.2.1, with the parameters “‐‐library-type fr-secondstrand ‐‐multi-read-correct ‐‐max-multiread-fraction 1.0 ‐‐no-length-correction ‐‐max-bundle-frags 1000000000”) (Trapnell et al. 2010) and the curated list of siRNA-producing loci. Differential expression analyses were carried out with CuffDiff (v2.2.1, with the parameters “‐‐library-type fr-secondstrand ‐‐no-length-correction ‐‐multi-read-correct ‐‐total-hits-norm ‐‐max-bundle-frags 1000000000”) (Trapnell et al. 2012). For all comparisons of reads mapping to siRNA-producing loci, except for the *hen1*/total-sRNA comparison, reads were normalized to the total number of reads mapping to the genome (specified by the “‐‐total-hits-norm” flag). Supporting the use of this simple normalization strategy, which normalized for only sequencing depth, the total number of normalized reads mapping to miRNAs was comparable for all data sets prepared in the same library batch—a result expected from properly normalized libraries from these samples, because the mutants were not expected to substantially influence the total levels of intracellular miRNAs. We found, however, that this simple normalization strategy was not sufficient for samples prepared in different library batches, and thus devised an alternative strategy to enable comparison of results from the *hen1* and total-sRNA samples, which were made in two separate batches. For this comparison, counts for each siRNA locus were enumerated with CuffDiff (output file genes.read_group_tracking), and these counts were then passed on to DESeq2 (parameters as described above for the miRNA analyses) with size factors manually set to normalize each sample to the total reads mapping to the cohort of abundant, Ago1-enriched, Dora-insensitive miRNAs (as defined above). For each plot, loci were filtered to retain those with one or more read in all samples, and a coefficient of variation of <1.0 for the three RPKM values from each clonal line for a genotype.

### Assessing Ago2 relative enrichments

Reads for individual miRNAs in wild-type cells for each total-sRNA and periodate-treated sample were normalized by dividing by the total number of reads mapping to miRNAs and multiplying this value by 1,000,000. The normalized reads for the three clonal lines for each genotype were then averaged, and the average for each miRNA in the sodium-periodate treated samples was divided by the average for that miRNA in the total-sRNA samples.

### RNA-seq

RNA-seq samples were prepared using the NEXTflex Rapid Directional mRNA-seq Kit (Bioo Scientific). Samples first were enriched for mRNAs using NEXTflex Poly(A) Beads (Bioo Scientific), and the enriched mRNAs were fragmented, synthesized into cDNA, ligated to adaptors, and PCR amplified according to the manufacturer's protocol. Samples were then sequenced on an Illumina HiSeq platform with 50-nt single-end reads. Reads were aligned to the genome, mapped to siRNA-generating loci, and analyzed as described above for the sRNA-seq reads that mapped to siRNA-generating loci but with the command “fr-firststrand” to account for the opposite strandedness of the libraries.

### Periodate treatment

RNA was oxidized in a solution of 25 mM borax (VWR), 25 mM boric acid (Sigma), and 25 mM of sodium (meta)periodate (Sigma Aldrich) for 30 min at room temperature. Oxidation was quenched with glycerol (to a final concentration of 17%), and the reaction was ethanol precipitated. Pellets were resuspended in a solution of 60 mM borax, 60 mM boric acid and the oxidized base was β-eliminated through the addition of NaOH to a final concentration of 50 mM NaOH and incubation at 45°C for 90 min. Samples were then ethanol precipitated.

### Immunoprecipitations

Ago1 was isolated using a FLAG-GST-tagged fragment of TNRC6B (Hauptmann et al. 2015). Pelleted cells were lysed in NET buffer (50 mM Tris, pH 7.5, 150 mM NaCl, 5 mM EDTA, 0.5% NP-40, 10% glycerol, supplemented with protease inhibitors (complete, mini, EDTA-free protease inhibitor tablets, Sigma Aldrich); 100 µL buffer per ∼1.5 million cells) on ice at 4°C for 20 min. Lysate was then clarified by centrifugation at 15,000*g* for 20 min. Anti-FLAG M2 Magnetic beads (Sigma Aldrich, 25 µL per 100 µL of lysate) were washed once with PBST (1xPBS supplemented with 0.02% Tween-20), and resuspended in 500 µL of PBST per each 25 µL beads. The TNRC6B fragment was then coupled to the washed beads by adding 12.5 µg of fragment per 25 µL of beads and rotating for 2 h at 4°C. Following coupling, beads were washed three times with PBS, and then the clarified lysate was added and the slurry was rotated at 4°C for 3 h. The beads were then washed four times with NET buffer and once with PBS before the RNA was eluted with TRIzol.

For immunoprecipitation of FLAG-tagged Ago2, cells were lysed in TBS-N (10 mM Tris, pH 7.5, 150 mM NaCl, 0.5% NP-40, supplemented with protease inhibitors (complete, mini, EDTA-free protease inhibitor tablets, Sigma Aldrich); 500 µL buffer per ∼10 million cells) on ice at 4°C for 20 min. Lysate was then clarified by centrifugation at 15,000*g* for 20 min and added to Anti-FLAG M2 Magnetic beads (25 µL per 500 µL of lysate) that had been washed twice with PBST. After rotation at 4°C for 3 h, the beads were washed three times with 200 µL of PBST, and RNA was eluted with TRIzol.

For protein analyses accompanying all immunoprecipitations, aliquots of beads were eluted in 2× NuPAGE LDS sample buffer (Life Technologies) with 0.1 M DTT. Aliquots of the input and flow-through fractions were also prepared in 1× NuPAGE LDS sample buffer supplemented with 0.1 M DTT.

### Protein expression and purification

The FLAG-GST-tagged fragment of TNRC6B was expressed and purified as described previously with slight modifications (Hauptmann et al. 2015). An expression construct for FLAG-GST-TNRC6B(599-683) (a gift from Gunter Meister) was transformed into BL21 cells [Rosetta 2(DE3) cells, Fisher Scientific]. A single transformant was used to seed an overnight culture in LB supplemented with 100 µg/mL Ampicillin (Gold BioTechnology) and 50 µg/mL Chloramphenicol (Gold BioTechnology), and 15 mL of the overnight culture was then used to seed each 1.5 L of LB supplemented with 100 µg/mL Ampicillin and 50 µg/mL Chloramphenicol. Once cultures reached an OD_600_ of ∼0.6, IPTG (Fisher Scientific) was added to a final concentration of 0.4 mM. After incubating overnight at 18°C with shaking, cultures were pelleted by spinning at 5000*g* for 20 min.

Pellets were resuspended in PBS (50 mL per 3 L of overnight culture) and lysed via sonication (50% power, five rounds of six cycles of 20 sec on, 20 sec off). Lysate was clarified by centrifuging 30 min at 25,000*g*, and then added to glutathione beads (Fisher Scientific) that had been washed once with PBS (1.5 mL per 3 L of overnight culture), and the slurry was rotated for 2 h at 4°C. The beads were then pelleted by spinning at 700*g* for 2 min, most of the supernatant was removed, and this reduced-volume slurry was then poured into a column, washed with 20 column volumes of PBS, and eluted with four column volumes of elution buffer (10 mM reduced glutathione, 20 mM Tris, 1 mM DTT in PBS). The eluate was further purified by anion-exchange chromatography on a Resource Q column (GE Healthcare) preequilibrated in buffer A (20 mM Tris pH 8.0, 25 mM NaCl, 1 mM DTT, and 5% glycerol). FLAG-GST-TNRC6B was eluted off the column with a linear gradient of buffer B (20 mM Tris pH 8.0, 1 M NaCl, 1 mM DTT, and 5% glycerol). Fractions with the greatest A_280_ signal were pooled and concentrated with buffer exchange back into buffer A using centrifugal filtration (Amicon Ultra 10 KDa cutoff, Fisher Scientific). Concentration of the protein was estimated by A_280_ (Nanodrop 2000, Thermo Scientific), and aliquots were flash frozen and stored at −80°C.

### Northern blots

Total RNA (5–10 µg per lane) was resolved on a 20% polyacrylamide urea gel and transferred to a Hybond-NX membrane (GE Healthcare) with a semi-dry transfer apparatus (Bio-Rad). Transferred RNA was crosslinked to the membrane by incubation with EDC [*N*-(3-dimethylaminopropyl)-*N*′-ethylcarbodiimide; Thermo Scientific] diluted in 1-methylimidazole for 1–2 h at 65°C, and then the membrane was blocked with Ultrahyb-Oligo (Life Technologies) for >10 min. Blots were probed overnight with either DNA or LNA radiolabeled oligonucleotide probes (Supplemental Table S5) suspended in Ultrahyb-Oligo, and results were visualized on a phosphorimager (Typhoon FLA 7000) and quantified using ImageQuant TL (v8.1.0.0). A step-by-step protocol can be found at http://bartellab.wi.mit.edu/protocols.html.

### Western blots

Proteins were resolved on a polyacrylamide gel (NuPAGE Bis-Tris 4%–12% gel, Life Technologies) using a Bolt Mini Gel Tank (Thermo Fisher Scientific) and MOPS running buffer (Life Technologies) as per the manufacturer's protocol. Proteins were then transferred to a PVDF membrane (Life Technologies) in transfer buffer (Life Technologies) using the Bolt Mini Gel Tank, according to the manufacturer's protocol. Membranes were blocked by incubation with 5% milk in PBST for 1 h at 4°C, and then incubated overnight at 4°C with primary antibody diluted in 5% milk in PBST. The following day, blots were washed with PBST for 5 min four times, incubated for 1 h shaking at room temperature with secondary antibody diluted in PBST, and then washed again with PBST for 5 min four times. Blots were visualized using the ECL Prime Western Blotting Detection Reagent (Thermo Fisher Scientific) according to the manufacturer's protocol, and imaged using a Bio-Rad ChemiDoc MP.

For primary antibodies, dilutions were 1:1000 for anti-Ago1 (Abcam, ab5070) and 1:2000 for anti-FLAG (Sigma Aldrich, #F1804). For secondary antibodies, dilutions were 1:10,000 for both HRP-conjugated anti-mouse and anti-rat IgG (Sigma Aldrich).

## DATA DEPOSITION

All sequencing data generated in this study have been submitted to the NCBI Gene Expression Omnibus (GEO; http://www.ncbi.nlm.nih.gov/geo/) under accession number GSE163938.

## SUPPLEMENTAL MATERIAL

Supplemental material is available for this article.

## Supplementary Material

Supplemental Material
