# Supplementary material for: Ago2 protects Drosophila siRNAs and microRNAs from target-directed degradation, even in the absence of 2′-O-methylation
Source: RNA. 2021 Jun;27(6):710–24. doi: 10.1261/rna.078746.121 (PMC8127995; doi:10.1261/rna.078746.121)
Supplement: Supplemental Material [file supp_27_6_710__DC1.html]

Ago2 protects Drosophila siRNAs and microRNAs from target-directed degradation, even in the absence of 2′-O-methylation — Ago2 protects Drosophila siRNAs and microRNAs from target-directed degradation, even in the absence of 2′-O-methylation — Supplemental Material 

# Ago2 protects *Drosophila* siRNAs and microRNAs from target-directed degradation, even in the absence of 2′-*O*-methylation

## Supplemental Material

- Supplemental\_Figure\_Legends.docx
- Supplemental\_Figures.docx
- Supplemental\_Table\_S1.xlsx
- Supplemental\_Table\_S2.xlsx
- Supplemental\_Table\_S3.xlsx
- Supplemental\_Table\_S4.zip
- Supplemental\_Table\_S5.xlsx
