# Supplementary material for: Ago2 protects Drosophila siRNAs and microRNAs from target-directed degradation, even in the absence of 2′-O-methylation
Source: RNA. 2021 Jun;27(6):710–24. doi: 10.1261/rna.078746.121 (PMC8127995; doi:10.1261/rna.078746.121)
Supplement: Supplemental Material [file supp_078746.121_Supplemental_Figure_Legends.docx]

**Supplemental Figure S1**. Analyses of periodate-treated and untreated small RNAs.

(*A*) Changes in miRNA levels observed upon loss of Dora in total-sRNA samples, as measured using sRNA-seq (replotted from Shi et al. 2020). Each point represents the mean from three biological replicates, as determined by DESeq. For both genotypes, each replicate used a different clonal line. Results for miRNAs significantly upregulated upon loss of Dora (adjusted *p* < 10^–8^) and the passenger strands of these Dora-sensitive miRNAs are shown in red and blue, respectively. (*B*) Enrichment in siRNAs observed in sRNA-seq libraries following periodate treatment. For each siRNA locus passing both the expression cutoff (RPKM greater than 5 in all samples) and reproducibility requirement (coefficient of variation of RPKM values for all three biological replicates within each genotype less than 1.0), mean read abundance (in RPKM) from the treated samples is plotted as a function of the mean abundance from the untreated samples. (*C*) Validation of periodate treatment, as indicated by correspondence between measured Ago2-enrichement values and values reported previously (Reichholf et al. 2019). For each miRNA, Ago2 enrichment was inferred by dividing the average fraction of miRNA reads corresponding to that miRNA in the periodate-treated libraries by the average fraction of miRNA reads corresponding to that miRNA in the untreated libraries. Spearman *R* for the comparison is shown.

**Supplemental Fig. S2**. Ago1 purification and analysis of Ago1-associated siRNAs.

(*A*) Purification of Ago1 using a FLAG-tagged fragment of TNRC6B (Hauptmann et al. 2015). Proteins from the input, flow-through (FT), and elution were analyzed on a western blot, probing for the indicated epitopes. In this co-IP, endogenous Ago1 was purified from cells that had been transfected with a plasmid expressing HA-Ago2, which enabled assessment of the purification of Ago1 away from contaminating Ago2. Percentages of each sample loaded onto each lane are specified in parentheses. (*B*) Depletion of Ago2-enriched miRNAs in Ago1 samples, as determined by sRNA-seq. Shown is the relationship between mean abundance in the Ago1 samples and mean abundance in the total-sRNA samples (three biological replicates for each genotype, each performed with a different clonal line). Points are colored by their Ago2-enrichment, as inferred by resistance to periodate oxidation (Supplemental Fig. S1C). (*C*) Sites predicted for select siRNAs (Czech 2008). For each site, the target is shown on top (oriented 5′ to 3′), and the siRNA is shown below (oriented 3′ to 5′). Watson–Crick base pairs are indicated with vertical bars, and G–U wobbles are indicated with slashes. (*D*) Changes in abundance of siRNAs mapping to annotated siRNA-generating loci observed upon loss of Dora in Ago1 samples. Otherwise, this panel is as in Fig. 1B.

**Supplemental Fig. S3**. Effects of *hen1* knockout on small-RNA levels and terminal methylation.

(*A*) Confirmation of reduced terminal methylation upon knockout of *hen1*. Samples from one WT, three *hen1*, and three *hen1/dora* clonal lines were either left untreated (–) or treated with sodium periodate and β-elimination (+), which removes the terminal nucleoside, unless its *cis* diol is blocked by either 2′-O-methylation or some other modification. Samples were resolved on a northern blot, which was probed for an siRNA (CG4068_1), an Ago2-enriched miRNA (miR-277), and an Ago1-enriched miRNA (miR-11). (*B*) Purification of FLAG-Ago2 from a *hen1* clone and a *hen1/dora* clone that had each been transfected with a plasmid expressing FLAG-Ago2. Proteins from the input, flow-through (FT), and elution of the IP were analyzed on a western blot, probing for the indicated epitopes. (*C*) Relationship between miRNA abundance in Ago2-associated samples from *hen1* cells with miRNA abundance in either periodate-treated samples or total-sRNA samples from WT cells. Plotted are mean values for the three biological replicates, each of which used a different clonal line. Pearson *R*^2^ for each correlation is shown. (*D*) Changes in abundance of Ago2-associated siRNAs mapping to annotated siRNA-generating loci observed upon loss of Dora in *hen1* cells. Otherwise, this panel is as in Fig. 1B. (*E*) Northern blot quantifying the abundance of two siRNAs (CG4068_1 and CR18854_1, previously known as esi-2.1 and esi-1.2, respectively) and a miRNA (miR-277) upon loss of Hen1. Each sample was from total sRNA collected from an independently derived clonal line. Each band intensity was normalized to that of the corresponding loading control (miR-11) and then to the average band intensity for all wild-type (WT) samples, yielding normalized relative intensities, shown for CG4068_1, CR18854_1, and miR-277.

**Supplemental Fig. S4**. Methylation does not protect an Ago1-loaded miRNA from TDD.

(*A,B*) Two additional replicates of Fig. 4A. (*C*) Quantification of the results in Fig. 4A and *S4A,B*. Otherwise, this panel is as in Fig. 4C. (*D*) TDD of endogenous miR-7 despite loss of Nibbler. Relative miRNA levels in each cell line were monitored on a northern blot, probed for miR-7, as well as miR-34, a known substrate of nibbler, and miR-11, which served as a loading control. The levels of both miR-34 and miR-7 relative to their average levels in wild-type cells are shown beneath their respective bands. The increased level of miR-34 observed upon loss of Dora suggested that this miRNA might also be a TDMD substrate. Supporting this suggestion, our sRNA-seq analyses indicated that miR-34 increased 1.7-fold (*p* = 0.00093) upon loss of Dora, despite failing to pass our more stringent threshold for confident identification of Dora-sensitive miRNAs (Fig. S1A). (*E*) The replicate of Fig. 4B.
